# Eine bessere Schule in post-pandemischen Zeiten? Differenzierte Gestaltungswünsche bei Wiener Schüler_innen

**DOI:** 10.1007/s11614-023-00513-7

**Published:** 2023-04-18

**Authors:** Ursula Holtgrewe, Irina Vana, Martina Lindorfer, Carmen Siller

**Affiliations:** 1 Zentrum für soziale Innovation, Arbeit und Chancengleichheit, Linke Wienzeile 246, 1150 Wien, Österreich; 3 University of Vienna, Wien, Österreich

**Keywords:** Schulreform, Soziale Ungleichheit, Schulschließung, Bildungskapital, School reform, Social School closures, Educational capital

## Abstract

Die Pandemie hat im österreichischen Schulsystem wie in anderen Ländern ein umfassendes Realexperiment ausgelöst, bestehende Ungleichheiten vertieft und neue Probleme aufgeworfen. Im Artikel werden, auf Basis einer Schüler_innenbefragung 2020, Vorstellungen und Erfahrungen von Schüler_innen über die Voraussetzungen guter und inklusiver (digitaler) Lernbedingungen thematisiert. Sowohl Herausforderungen des Lernens als auch Bedarfe und Ideen für die Neugestaltung von Schule sind eingebettet in ungleiche soziale Ressourcen und Kapitalien. Es wird gezeigt, wie die Wünsche der Schüler_innen und deren schichtspezifisch geprägte Lernstrategien ineinandergreifen und warum das klassische Digitalisierungsprogramm der Ausstattung mit Hardware und der Schulung digitaler Skills bei der Frage, welche Reformen im Schulsystem zur Überwindung von Ungleichheiten nötig sind, an seine Grenzen stößt.

## Abstract (en)

As in other countries, the pandemic has triggered a comprehensive real-world experiment in the Austrian school system, deepening existing inequalities and raising new issues. This article explores students’ perceptions and experiences of the prerequisites for good and inclusive (digital) learning conditions. The findings are based on a student survey conducted in 2020. It is argued, that challenges of learning during school closures and the needs and ideas for reforming the school system are both shaped by unequal social resources and capitals. The article shows how students’ desires for reforms in school and their class-specific learning strategies intertwine and explains why the classical digitalization program of equipping students with hardware and teaching digital skills reaches its limits when it comes to the question of which reforms in the school system are necessary to overcome inequalities.

## Einleitung

Die Pandemie hat im Schulsystem ein umfassendes Realexperiment ausgelöst: In einer bislang heterogenen Digitalisierungslandschaft (bmwf 2018) haben sich Schulen, Lehrer_innen und Schüler_innen in einer knappen Woche auf digitalisiertes, räumlich distanziertes Lernen umstellen müssen. Elemente des „traditionelle“ Unterrichts wie Feedback, Erklärungen und Möglichkeiten zur Kooperation blieben im digitalen Raum oftmals unrealisiert oder mussten erst neu entwickelt werden (Porsch und Porsch 2020, S. 103). Damit standen die Wertigkeiten von im Präsenzunterricht erprobten Praktiken und Lernformaten ein Stück weit zur Diskussion, wobei sowohl informelle Lerngelegenheiten als auch Möglichkeiten, über Standards und Erwartungen zu verhandeln, nach Bildungskapital ungleich verteilt waren (Holtgrewe et. al. 2021).

Krisen verändern bestehende Handlungsroutinen. Sie machen damit die Routinen und Normen des „Normalbetriebs“ bewusst und zeigen, unter welchen Voraussetzungen diese funktionieren (Grunert et al. 2022, S. 90). Die Veränderung zeigt so Probleme und Potenziale auf. Wenige Studien gingen bisher über die Feststellung der Verstärkung ungleicher Bildungschancen hinaus, um Veränderungspotentiale im Schulsystem zu ergründen, die jenseits der klassischen Digitalisierungsperspektive („mehr Computer und passende Hardware“) liegt (Helm et al. 2021). Diese rücken wir mit der Frage nach den Gestaltungswünschen der Schüler_innen an die Schule und das Lernen in den Fokus. Wir fragen, wie deren sozial ungleiche Voraussetzungen und Erfahrungen beim Lernen auf Distanz, deren Perspektiven auf Innovations- und Veränderungspotentiale des Schulsystems prägten.

## Formale Bildung und soziale Ungleichheit

Das Bildungssystem ist eine Struktur, durch welche soziale Differenz aufrechterhalten und sozialer Status reproduziert werden (Steiner 2019; Bourdieu und Passeron 1971; El-Mafaalani 2020). Erfolg und Misserfolg im System werden zwar „Leistung“ zugerechnet, aber davon mitbestimmt, wie gut Schüler_innen die dominanten kulturellen und sozialen Normen des Systems verstehen, interpretieren und anwenden können (Bourdieu und Passeron 1971). Diese Kompetenzen werden stark im Zuge der primären Sozialisation vermittelt und sind durch das soziale Umfeld geprägt.

Der Einfluss, den das persönliche Umfeld auf Lernstrategien und -erfahrungen nimmt, kann nach Bourdieu mit dem Konzept der kulturellen Reproduktion (Nash 1990, S. 440) oder nach Boudon (1974) mit jenem schichtabhängiger Bildungsstrategien und -aspirationen gefasst werden (Rutter 2018). Der in Bezug auf die Fragestellung relevante primäre Effekt der sozialen Herkunft begründet, dass Schüler_innen aus höheren Sozialschichten durch ihrer Erziehung eher Fähigkeiten entwickeln, die in der Schule vorteilhaft sind (vgl. Becker und Lauterbach 2016, S. 16). Das macht Bildungssysteme umso ungleicher, je mehr die Herkunftsfamilie in die Ausbildung involviert ist (Becker und Lauterbach 2016, S. 27). In Österreich gelingt der Ausgleich von ungleichen Bildungschancen nur in geringem Maße (Statistik Austria 2018). Ein Grund dafür liegt in der Tradition der Halbtagsschulen, durch welche soziale Unterschiede in den Ressourcen der Familien für den Lernerfolg von Kindern relevanter werden (Solga und Dombrowski 2009, S. 21).

Während Boudon vor allem auf die Bildungsstrategien und -aspirationen der Schüler_innen fokussiert, analysiert Bourdieu das Setting Schule und argumentiert, dass Lehrpläne, Bildungsgrundsätze und Bewertungskriterien des Schulsystems dem Verhalten, Werte- und Relevanzsystem bildungsnaher Mittelschichten entsprechen (Grenfell 2004, S. 50) wodurch Schüler_innen, deren Umgangsformen den dominanten Praxisformen in der Schule entsprechen, von Lehrkräften und Bezugspersonen eher eine positive Reaktion auf ihr Lernverhalten erhalten (Fredricks et al. 2004) und sich im System leichter orientieren. Mit anderen Worten: Nicht nur die ungleiche Verteilung materieller Ressourcen oder eine geringere Unterstützung zu Hause machen den Unterschied, wie gut Kinder mit den Anforderungen des Schulsystems umgehen können. Auch die Anforderungen von Schulen und das sozial geprägte Lernverhalten reproduzieren soziale Ungleichheiten und bieten unterschiedliche Entwicklungsmilieus, die verschiedene Kompetenzen fordern und fördern (Maaz 2020). Die hier gestellte Frage nach den Wünschen der Schüler_innen trägt dazu bei, über die Diagnose der Benachteiligungen hinauszugehen und die Gestaltungsideen der Betroffenen in den Diskurs einzubringen.

### Auswirkungen der Schulschließung

Mit der Schließung der Schulen wurde die Verantwortung zur Unterstützung und Organisation des Lernalltags der Schüler_innen weitgehend in die Hand der Familien bzw. einzelnen Schüler_innen gelegt. Damit haben sich ungleichen Bildungschancen vertieft und zu einem verstärkten Unterstützungsbedarf geführt. Ein systematischer Review der Studien im deutschsprachigen Raum zeigt, dass vor allem die Belastungen von Schüler_innen aus ärmeren, einfacher gebildeten Familien zunahmen (Helm et al. 2021). Neben mangelnden Unterstützungsmöglichkeiten der Eltern, fällt ins Gewicht, dass Lehrkräfte Nachfragen im Distanzunterricht weniger aufgreifen konnten (Schwabe und Lindner 2021, S. 47) und, dass Erwartungen der Lehrkräfte in Bezug auf die Entwicklung von benachteiligten Schüler_innengruppen einer positiven Leistungsentwicklung dieser Schüler_innen in der Art einer *self-fulfilling prophecy *entgegenstanden (ebda., S. 58). Die Schulkostenstudie 2020 zeigte zudem, dass sich mit der Pandemie die psychische Gesundheit etwa jedes dritten Kindes verschlechtert hat, womit die emotionalen Voraussetzungen zum Lernen unter Druck geraten sind (Schönherr et al. 2021).

Helm et al. (2021) identifizieren drei zentrale Faktoren für das Gelingen des Unterrichts auf Distanz und für die Aufrechterhaltung von Lernbemühungen. Dazu zählen häusliche Ressourcen, schüler_innenbezogene Merkmale, wie deren Selbstständigkeit und Motivation (ebd., S. 241), sowie Qualitätsmerkmale des Unterrichts und der Lehrkräfte. Wir fokussieren in diesem Artikel auf das Zusammenspiel der schüler_innenbezogenen Merkmale, Lernerfahrungen und -praktiken und die häuslichen Ressourcen (z. B. die elterliche Unterstützung).

## Vorgehen und Datensatz

Anhand der Ergebnisse der Befragung „Lernen im Ausnahmezustand“^1^, die zwischen April 2020 und Juni 2020 durchgeführt wurde, untersuchen wir, wie sich die Lernerfahrungen von Schüler_innen nach deren Bildungskapital unterscheiden und wie das ihre Erwartungen an ein besseres Lernumfeld prägte. Berücksichtigt werden auch Einflüsse des Geschlechts, Alters und des Schultyps.

Die Lernerfahrungen der Schüler_innen wurden zu zwei Zeitpunkten erfasst: Während des ersten Lockdown, als die Mehrzahl der Schüler_innen zu Haus blieb, und nach der Teilöffnung der Schulen Ende Mai 2020, als Schüler_innen alternierend, an zwei bis drei Tagen die Woche die Schule besuchten. Die vor dem Hintergrund der Erfahrungen formulierten Gestaltungswünsche thematisieren wir im abschließenden Kapitel. Diese wurden im dritten Fragebogen im Juni 2020 erfragt, als der Lehrbetrieb wieder weitestgehend in Präsenz stattfand.

### Datensatz

Mittels online-Fragebogen wurden Schüler_innen von vier Volksschulen, vier Neuen Mittelschulen, einem Gymnasium (AHS), einem Oberstufenrealgymnasium und einer berufsbildenden Schule in Wien befragt. Über drei Befragungswellen hinweg haben 503 Schüler_innen mindestens einmal einen Fragebogen ausgefüllt. Den ersten Fragebogen, haben 349 Schüler_innen beantwortet. An der zweiten Befragung (Welle 2), nahmen 185 Schüler_innen teil. An der Bilanzbefragung (Welle 3) beteiligten sich 90 Schüler_innen. Einen genauen Überblick über die soziodemographischen Merkmale der teilnehmenden Schüler_innen der unterschiedlichen Wellen bietet Tab. 1 im Anhang.

Alter, Geschlecht und soziale Herkunft prägen die Antwortbereitschaft und Erreichbarkeit der Schüler_innen. Über alle Befragungswellen hinweg haben mehr Schülerinnen als Schüler an der Befragung teilgenommen. Auch war bereits während der ersten Befragung der Großteil der Teilnehmenden älter als 14 Jahre (63 %). Die Zahl der Jüngeren sank im Juni, bei der zweiten Befragung, weiter. Bei der Verteilung der Schüler_innen nach Schultyp erweist sich ebenfalls ein Bias: Bereits in der 2. Welle nahmen nur mehr 10 NMS Schüler_innen an der Befragung teil. Von den Berufsschüler_innen beteiligten sich an der abschließenden Befragung nur noch 12 Personen. Schüler_innen, die zum Zeitpunkt der Schulschließung eine weiterführende Schule (AHS-Oberstufe oder Realgymnasium) besuchten, sind im Sample daher überrepräsentiert.^2^

Analog zu den Schultypen, beteiligten sich Schüler_innen aus höher qualifizierten Familien eher an der Befragung: Während von den Wiener Schüler_innen im Alter bis 19 Jahren 28 % mind. ein Elternteil einen akademischen Abschluss hatten, liegt der Anteil der Akademiker_innenkinder im Sample bei 46 % (vgl. Statistik Austria 2020a).

Ein direkter Bezug zwischen den drei Wellen kann wegen der Panelmortalität nicht hergestellt werden: 65 der Schüler_innen haben an der ersten und zweiten Welle der Umfrage teilgenommen, aber nur 28 Schüler_innen beantworteten alle drei Fragebögen.

### Operationalisierung von Bildungskapital, Lernerfahrungen und Wünschen

Das Bildungskapital der Familien wurden über die Qualifikation der Eltern mittels drei Variablen gemessen: Information zu den Berufen beider Eltern^3^, der Zahl der Bücher im Haushalt^4^, sowie anhand des höchsten Ausbildungsabschlusses des höher qualifizierten Elternteils.^5^ Differenziert wird zwischen (1) hoch qualifizierten bzw. akademischen Familien, in denen mindestens ein Elternteil einen akademischen Abschluss hat bzw. einen akademischen Beruf ausübt, (2) Familien mit höherer Qualifikation (höhere berufsbildende Schule, höhere technische Berufe) oder mit mittlerer Qualifikation, in denen mindestens ein Elternteil einen Lehrabschluss oder einen Maturaabschluss hat, sowie (3) Familien mit einfacher Qualifikation, in welchen weder Mutter noch Vater zumindest eine Lehre abgeschlossen haben.

Um die Erfahrungen der Schüler_innen mit dem Lernen auf Distanz zu erfassen, wurden ihre emotionale Situation beim Lernen (Freude am Lernen und Überforderung), Lernpraktiken (wie sie sich Hilfe holten) und inhaltliche Schwierigkeiten erfragt. Diese bilden unterschiedliche Dimensionen des Lernens ab, welches als soziokulturell eingebetteter kognitiver, verhaltensbezogener und affektiver Prozess beschrieben werden kann (vgl. Falk et al. 2016).^6^ Zusätzlich fragten wir die Schüler_innen direkt was es braucht, damit das Lernen gelingt.^7^ Die Antworten auf die offenen Fragen dienen im weiteren der Illustration der quantitativen Ergebnisse.

Die *Gestaltungswünsche der Schüler_innen*, wurden in der dritten Welle erfragt, an der jedoch nur 90 Schüler_innen teilnahmen. Die analysierten Aussagen der Schüler_innen stammen vor allem von Schüler_innen der AHS-Oberstufe bzw. des Realgymnasiums.

### Datenauswertung

Die Lernerfahrungen unterschiedlicher Schüler_innengruppen werden entlang von affektiven und verhaltensbezogenen Aspekten des Lernens beschrieben. Zur Darstellung der Einflüsse des Bildungskapitals auf die Lernerfahrungen und Lernpraktiken, sowie die Gestaltungswünsche, werden Unterschiedstests berechnet und Effektstärken angegeben.^8^

## Ergebnisse: Erfahrungen während der Pandemie und darauf aufbauende Wünsche

Ausgehend von der Frage, wie verschiedene Schüler_innengruppen das Lernen im Lockdown und während der Teilöffnung gefiel werden im Folgenden inhaltliche Schwierigkeiten und Lernstrategien der Schüler_innen während des Lockdowns genauer betrachtet. Der Zeitvergleich zeigt die ungleiche Wirkung der Schließung schulischer Lernräume und neuer Lernarrangements auf Lernerfahrungen und Wohlbefinden der Lernenden. Diese Überlegungen unterstützend folgt die Interpretation von Gestaltungswünschen, wobei nach Bildungskapital unterschieden wird.

### Lernerfahrungen und Lernpraktiken in der Pandemie

Die Mehrzahl der Schüler_innen (55 %) meinte, dass ihnen das Lernen zu Hause während des Lockdowns sehr oder eher gefällt. Nur ein Viertel lernte lieber in der Schule. Das entspricht den Befunden vergleichbarer Studien (vgl. Helm et al. 2021, S. 266; Schober et al. 2020). Die Sichtweise der Schüler_innen ist jedoch ambivalent. Je älter die Schüler_innen waren, desto differenzierter war ihr Meinungsbild. Mädchen über 14 Jahre gefiel das Lernen zu Hause trotz empfundener Belastungen besonders häufig (62 %)^9^. Das Wegfallen des sozialen Kontexts der Schule war für sie auch entlastend. Das gilt auch für Schüler_innen aus Familien mit niedrigem Bildungskapital, von denen 35 % meinten, dass sie weniger Kontakt mit den Mitschüler_innen schätzen. Insgesamt meinten das 20 %.^10^ Vermutlich fällt für diese Gruppen auch sozialer Druck und sozialer Vergleich in der Schule weg.

Die Freude am Lernen hängt eng mit der erfolgreichen Bewältigung der Aufgaben zusammen.^11^ Unter jenen Schüler_innen, die mit den Aufgaben sehr gut zurechtkamen, gaben 41 % an, dass ihnen das Lernen zu Hause sehr gut gefällt. Schüler_innen, die mit den Aufgaben eher nicht oder nicht zurechtkamen, meinten das nur zu 9 %. Inhaltliche Schwierigkeiten mit den Aufgaben lagen nach unserer Definition vor, wenn Schüler_innen sich bei den Hausaufgaben nicht auskannten, ihnen oft Erklärungen fehlten und sie ihre Aufgaben nicht gut erledigen konnten.^12^

**Figure 1:**
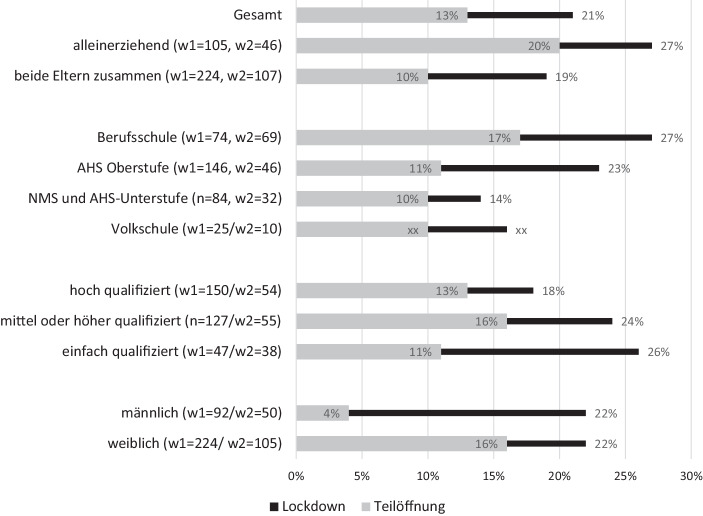

Das traf während des Lockdowns auf 21 % der Schüler_innen zu (vgl. Abb. 1). Die Lösung der digital übermittelten Aufgaben fiel Schüler_innen aus einfach qualifizierten Familien in der Tendenz schwerer. Auch ältere Schüler_innen und Berufsschüler_innen berichteten eher von Schwierigkeiten. Schüler_innen, deren Eltern getrennt leben, hatten ebenso häufiger inhaltliche Probleme.

Der Präsenzunterricht nach der Teilöffnung der Schulen hilft bei inhaltlichen Problemen, war für die im Schulsystem benachteiligten Gruppen jedoch besonders wichtig. Hatten im Lockdown insgesamt 21 % Schwierigkeiten mit der Aufgabenbewältigung, so berichten das nach der Teilöffnung nur noch 13 %. Vor allem Schüler_innen aus geringer qualifizierten Familien profitierten von der Schulöffnung (vgl. auch Schwabe und Lindner 2021, S. 47; Schober et al. 2021). Auch Buben bzw. junge Männer profitierten stärker von der Teilöffnung. Die Ergebnisse weisen auch darauf hin, dass mehr Unterstützung zu Hause, wenn etwa zwei Eltern sich bei der Unterstützung der Schüler_innen abwechseln konnten, das Lernen im Lockdown erleichterte.

Insgesamt berichtet ein Drittel der Schüler_innen im Lockdown von Überforderung (35 %). Schüler_innen einfach qualifizierter Familien waren mit 48 % besonders häufig überfordert. Von Schüler_innen aus hochqualifizierten Familien berichtete nur etwas mehr als ein Viertel, dass sie tendenziell überfordert waren.^13^ Die Gründe für die Überforderung können, wie die Aussagen der Schüler_innen in offen gestellten Nachfragen nahelegen, vielseitig sein: „*Weil die Aufgaben sehr intensiv sind und es kaum Abwechslung gibt. In der Schule ist nicht jede Stunde gleich intensiv, aber zuhause bekommen wir nur intensive Aufgaben*“, erklärt etwa ein Schüler der AHS-Oberstufe (18 Jahre, hochqualifizierte Familie). Das beschreibt eine, wie wir meinen, wichtige Belastungserfahrung beim Lernen zuhause. In der Schule kann man mal ganz aktiv, mal eher rezeptiv am Unterricht teilnehmen, ohne aus der Lernsituation herauszufallen. Man wird beim Lernen auf Distanz weniger von der sozialen Lernsituation getragen. Der Verlust dieses Lernrahmens trifft, wie unsere Daten zeigen, jene besonders, die weniger Unterstützung dabei haben, sich das Lernen selbst einzuteilen.

**Figure 2:**
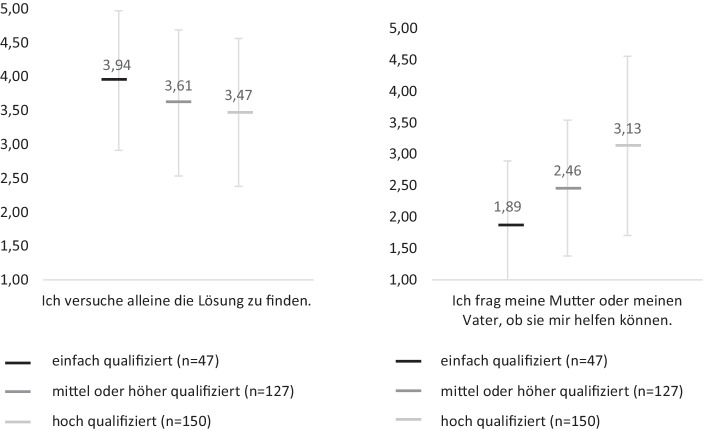

Abb. 2 zeigt unterschiedliche Lernstrategien der Schüler_innen nach Bildungskapital. Auf einer Skala von 1 (nie) bis 5 (immer) wurde gefragt, was die Schüler_innen taten, wenn sie mit einer Aufgabe nicht zurechtkamen. Während rund ein Viertel der Schüler_innen, deren Eltern einfach qualifiziert sind, Unterstützung der Eltern erbat,^14^ taten das Schüler_innen, deren Eltern hoch qualifiziert sind, viel häufiger (50 %). Kinder der Einfachqualifizierten versuchten häufiger, die Lösung alleine zu finden. Hilfe durch Schulkolleg_innen oder Lehrer_innen wurde von dieser besonders belasteten Gruppe nicht häufiger genutzt als von den anderen, konnte also ohne den gemeinsamen Unterricht den Bedarf nicht kompensieren.

Der Aufwand von Schüler_innen, ihren Lerntag zu planen, war bei fehlender Unterstützung zu Hause bedeutend höher. 59 % der Schüler_innen aus Familien mit einfacher Qualifikation meinten auf die Frage, wie sie entschieden, wann welche Aufgaben zu machen seien, dass sie bereits am Vortag planten. Das traf nur auf 38 % der Schüler_innen aus akademischen Familien zu. Diese konnten eher mit der Hilfe der Eltern rechnen, um den Tag zu strukturieren und waren daher auch freier darin, „nach Lust und Laune“ zu planen oder nicht. Selbstorganisation und Planung ist also für die Kinder der Einfachqualifizierten notwendiger, bleibt aber herausfordernd: 15*„Zuhause lernen gefällt mir gar nicht!! Es ist zwar angenehm, weil ich daher länger schlafen kann, aber das hilft mir nicht beim Stoff, ich komme ohne Hilfe nicht weiter!!“ *konkretisiert eine Schülerin (16 Jahre, einfach qualifizierte Eltern).

Diese differenzierte Sicht der Schüler_innen auf das Lernen in der Schule und zu Hause, zeigt Erfahrungen des Mangels, den die erwähnten Risikogruppen im Schulsystem alltäglich erleben. Sie behelfen sich mit mehr Planung, aber wissen, dass sie mit Hilfe und sozialem Kontakt besser und entspannter lernen können. Das zeigt sich auch in den Wünschen an die Gestaltung des schulischen Lernumfelds.

### Gestaltungswünsche an ein besseres Lernumfeld

Gestaltungswünsche an das Lernen und den Alltag in der Schule werden vor dem Hintergrund dieser differenzierten und ambivalenten Erfahrungen der Schüler_innen interpretiert, wobei wir die Unterschiede nach Bildungskapital herausgreifen. Unsere Auswahl an Items enthält reformpädagogische und eher konventionelle digitalisierungsbezogene Vorschläge, die wir sowohl theoriegeleitet als auch aus den Daten der beiden vorherigen Befragungswellen entwickelt haben.

**Figure 3:**
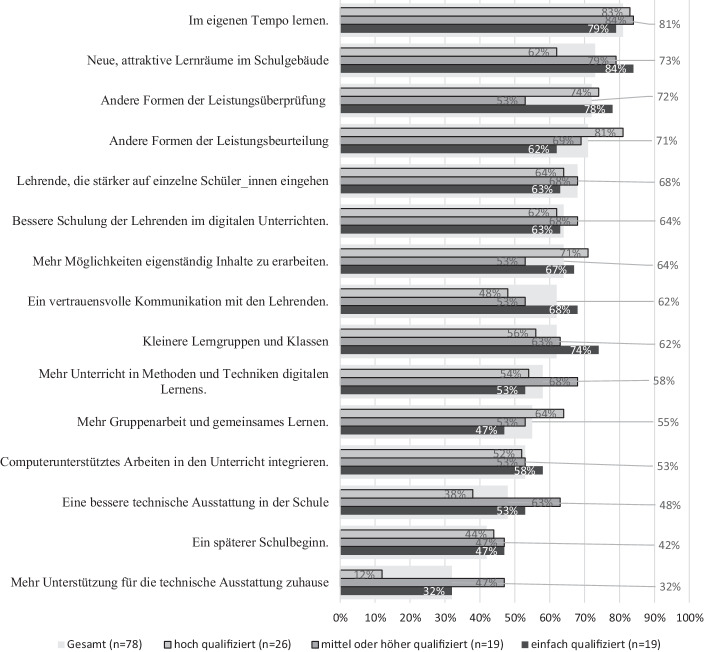

Abb. 3 zeigt eine Liste der von den Schüler_innen angeführten Wünsche für eine Neugestaltung der Lernensituation und -umgebung nach Bildungskapital der Eltern.^16^ Die Möglichkeit, im eigenen Tempo zu lernen, rangiert mit 79 % der Nennungen ganz oben.^17^

Spannend ist auf Rang 2 der Wunsch nach attraktiven Lernräumen in der Schule. Hier stimmen 73 % zu. Der Wunsch rangiert bei den Gruppen am höchsten, die durch die Schulschließung besonders belastet waren. Von den Schüler_innen aus einfachqualifizierten Familien bewerteten 84 % die Schaffung neuer Lernräume als wünschenswert. Überraschend ist das nicht: Wir sehen auch in anderen Befunden, dass die Schulschließungen für jene Schüler_innen besser auszuhalten waren, die zuhause ein attraktiveres Umfeld haben. Kreativräume, Leseecken, Gruppenräume und multifunktionale Räume sollten also, sowohl im Lichte der Förderung selbstbestimmten Lernens als auch der Resilienz von Schulen und Bildungs-Infrastrukturen, vermehrt erschlossen und auch für den Normalbetrieb ausgebaut und genutzt werden.

Im Ranking folgt der Wunsch nach Lehrenden, die stärker auf einzelne Schüler_innen eingehen mit 68 % Zustimmung. Nach der Bildungsschicht sehen wir hier keine nennenswerten Unterschiede.

Eine bessere Schulung der Lehrenden für das digitale Unterrichten wünschen sich 64 % der Befragten. Schüler_innen aus mittel- bzw. höher qualifizierten Familien äußern den Wunsch in der Tendenz häufiger (68 %). Diese Gruppe wünscht sich auch überproportional oft eine bessere Schulung der Schüler_innen im digitalen Lernen (68 %).

64 % der Schüler_innen bewerten andere Formen der Leistungsüberprüfung anstelle von Tests und Schularbeiten als wichtige Innovation. Solche Formate wurden während der Pandemie zum Teil praktiziert. Betrachtet nach Bildungskapital ist die Zustimmung bei Schüler_innen aus einfachqualifizierten Familien zu dieser Maßnahme am höchsten (74 %). Der Wunsch nach anderen Formen der Leistungsüberprüfung spiegelt einerseits die Zustimmung zu einer Pädagogik wider, die die etablierte „Fehlerkultur“ überwindet und die positiven Leistungen der Schüler_innen gegenüber den Schwächen in den Fokus nimmt. Daher findet diese Maßnahme auch bei Schüler_innen aus dem akademischen Milieu viel Zuspruch. Andererseits zeigt sich darin der Wunsch, Leistung in ihren unterschiedlichen Facetten sichtbar und bewertbar zu machen. Das ist für Schüler_innen aus Familien mit einfacher Qualifikation entscheidend.

Gleichermaßen wichtig sind den Schüler_innen eine vertrauensvolle Kommunikation mit den Lehrenden (62 %) und kleinere Klassen (61 %). Beides ist vor allem Schüler_innen aus niedriger qualifizierten Familien wichtig. 68 % von diesen – gegenüber 48 % der Schüler_innen aus hochqualifizierten Familien – wünschen sich hier eine Verbesserung. Wir vermuten, dass Schüler_innen aus niedriger qualifizierten Familien potenziell einen stärkeren Mangel erleben oder mehr soziale und habituelle Distanz zu den Lehrenden wahrnehmen.

Kleinere Klassen werden auch überproportional häufig von Schüler_innen aus einfachqualifizierten Familien (74 %) gewünscht. Schüler_innen aus hochqualifizierten Familien sahen hier nur zu 56 % einen Bedarf. Kleinere Lerngruppen sind demnach, ebenso wie die Schaffung attraktiver Lernräume, verstärkt ein Thema für benachteiligte Gruppen.

Es folgen die Möglichkeiten, eigenständig Lernstoff zu erarbeiten mit 64 %. Darauf legen sowohl Schüler_innen aus hoch qualifizierten als auch die aus niedriger qualifizierten Familien mehr Wert als jene aus der Mitte.

Je 55 % der Befragten wünschen sich mehr Gruppenarbeit und andere Formen der Leistungsbeurteilung. Beides sind Themen, die vor allem Schüler_innen aus hochqualifizierten Familien wichtig sind (65 %). Nur 47 % der Schüler_innen aus einfach qualifizierten Familien wünschen sich mehr Gruppenarbeit. Auch Wunsch nach alternativen Formen der Leistungsbeurteilung bleibt ein Thema der Schüler_innen aus hochqualifizierten Familien. Diese Gruppe kann durch ihre habituelle Bildungsnähe damit rechnen, auch mit neuen und offenen Lern- und Beurteilungsformaten zurechtzukommen (Sertl 2007). Schüler_innen aus niedriger qualifizierten Familien sehen hingegen in neuen Beurteilungsformen seltener einen Fortschritt.

Die Kernthemen der Digitalisierung in der Schule liegen in der Rangliste der Wünsche der Schüler_innen eher hinten. 53 % wünschen sich, dass computerunterstütztes Arbeiten stärker in den Unterricht integriert wird. Nach Bildungsgrad der Familien gibt es hier wenig Unterschiede. Eine bessere technische Ausstattung in der Schule wünschen 48 %, wobei Schüler_innen aus mittel- bzw. höherqualifizierten Familien (63 %) das öfter wünschen als jene aus hochqualifizierten Familien (38 %).

Nur 32 % wünschen Unterstützung für die technische Ausstattung zuhause. Diese spielt zwar in in der politischen Digitalisierungsstrategien 2023 mit der geplanten Verteilung von Tablets an Schüler_innen der 5. Schulstufe eine zentrale Rolle, ist aus Sicht der Lernenden aber weniger relevant. Vor allem Schüler_innen aus mittel- bzw. höherqualifizierten Familien nennen diesen Wunsch (47 %) und befürworten auch eine bessere Ausstattung der Schule mit Computern häufiger (63 %). Nur jede_r Vierte aus einer einfach qualifizierten Familie stimmt hier zu, aus den hochqualifizierten Familien sind es 12 %.

Zusammenfassend zeigt sich, dass Schüler_innen aus einfach qualifizierten Familien Unterstützungsstrukturen an der Schule, wie etwa attraktive Räume, kleinere Lerngruppen, bessere Kommunikation mit Lehrenden und Feedback durch diese besonders befürworten. Ein klares Thema der begünstigten Gruppen ist die Eigenständigkeit. Digitalisierungsthemen wird insbesondere von Schüler_innen aus mittelqualifizierten Familien ein hoher Wert zugemessen.

## Schlussfolgerungen

Insgesamt zeichnet sich ab, dass die Schulschließung soziale Ungleichheiten verschärft hat. Wir schlussfolgern, dass alle Schüler_innen vom Schulsetting profitieren, um eine Alltagsroutine herzustellen, dass aber jene, die zu Hause weniger Unterstützung erhalten können, davon besonders profitieren.

Die ambivalente Haltung der Schüler_innen zum Lernen während des Lockdowns unterstreicht den Bedarf nach einem sozialen Lernraum und einer inklusiveren Gestaltung dieses Lernraums. Die These von Sertl (2007), dass reformpädagogische Ansätze allgemein die Kinder der Mittelschichten weiter begünstigen, erfährt in der Sicht der Schüler_innen eine Differenzierung:

Schüler_innen aus den bildungsbenachteiligten Familien wünschen sich attraktive Räume zum selbstbestimmten Lernen, vertrauensvolle Kommunikation mit den Lehrenden und kleinere Lerngruppen – also Freiräume *und* intensivere Betreuung. Mit den Schüler_innen aus bildungsbegünstigten Familien teilen sie den Wunsch nach anderen Formen der Leistungsüberprüfung und nach Möglichkeiten eigenständiger Arbeit. Beide Gruppen möchten auf vielfältigere Weise lernen und zeigen, was sie gelernt haben – und besonders die Bildungsbenachteiligten wollen und brauchen dazu die geeignete Umgebung und persönliche Unterstützung. Mit Blick auf den höheren Planungsaufwand, den Schüler_innen aus einfach qualifizierten Familien im Lockdown hatten, scheint ihnen mehr Selbstorganisation ohne Unterstützung vermutlich eher als Last denn als Entfaltungschance. Ein anderer Teil der reformpädagogischen Agenda ist tatsächlich eher Thema der Schüler_innen aus bildungsbegünstigten Familien: Andere Formen der Leistungs*beurteilun*g sowie Gruppenarbeit und gemeinsames Lernen.

Schüler_innen aus mittelqualifizierten Familien stimmen am meisten einer klassischen, konventionellen Digitalisierungsagenda zu. Auch im eigenen Tempo lernen zu können, ist für sie besonders attraktiv. Eher reformpädagogische Themen wie eigenständige Arbeit oder andere Formen der Leistungsüberprüfung wünschen sie seltener. Wir vermuten, dass die Kinder aus mittelqualifizierten Familien am ehesten an familiale Erfahrungen mit quasi industriegesellschaftlich formalisierten und technisch gut ausgestatteten Qualifikations- und Aufstiegswegen anschließen. Diese in der Familie tradierte Erfahrung prägt auch die Wünsche und Erwartungen der Schüler_innen.

Das entstehende Meinungsbild zeigt, dass unterschiedlich benachteiligte und bevorzugte Gruppen auch unterschiedliche Erwartungen und Hoffnungen in Reform- und Digitalisierungsvorschläge setzen. Schüler_innen urteilen dabei weder nur nach Bedürfnissen noch nur nach Interessen. Sie überlegen, was sie wollen und brauchen, sowohl um ganz intrinsisch gut zu lernen, als auch, um dabei erfolgreich zu sein. Methodisch ist es mit einer kleinen und kompakten Befragung natürlich nur äußerst begrenzt möglich, Erfahrungen und Wünsche im Lichte von intersektional und biographisch konstituierten Aspirationen zu erfassen. Unsere Befunde zeigen aber an, dass es möglich und notwendig ist, über die Logik von Risikogruppen hinauszukommen und auch Gestaltungs- und Reformvorschläge, die Corona-„Lessons Learned“, nach ihren Ungleichheitsdimensionen auszuleuchten.

## Anhang

**Table 1:**

	Lockdown: April 2020		Teilöffnung: Mai 2020		Bilanz: Ende Juni 2020		Im Gesamt Sample	
	n	%	N	%	N	%	N	%
**Geschlecht**								
Weiblich	235	70	123	68	65	75	341	70
Männlich	100	30	59	32	22	25	145	30
*Gesamt*	*335*	*100*	*182*	*100*	*87*	*100*	*486*	*100*
Kann/will ich nicht sagen	14	–	3	–	3	–	17	–
**Alter**								
Bis 9 Jahre	15	4	9	5	3	3	22	4
10 bis 14 Jahre	115	33	59	32	19	21	148	29
Über 15 Jahre	219	63	117	63	68	76	333	66
**Schultyp**								
Volkschule	26	7	11	6	3	3	33	7
NMS	45	13	10	5	2	2	49	10
AHS Unterstufe	42	12	24	13	15	17	52	10
AHS Oberstufe	156	45	62	34	58	64	218	43
Berufsschule	80	23	78	42	12	13	151	30
**Bildungskapital**								
Einfach qualifiziert	52	15	43	26	21	28	92	20
Mittel qualifiziert	92	27	53	32	16	22	135	29
Höher qualifiziert	37	11	7	4	6	8	42	9
Hoch qualifiziert	156	46	63	38	31	42	190	41
*Gesamt*	*337*	*100*	*166*	*100*	*74*	*100*	*459*	*100*
Keine Angabe	12	–	19	–	16	–	44	–
**Mehrsprachig**								
Nein	181	53	73	44	33	46	223	56
Ja	161	47	94	56	39	54	173	44
*Gesamt*	*342*	*100*	*167*	*100*	*72*	*100*	*396*	*100*
Keine Angabe	7	–	18	–	18	–	41	–
**Alleinerziehend**								
Nein	234	67	130	72	69	79	339	68
Ja	114	33	51	28	18	21	157	32
*Gesamt*	*348*	*100*	*181*	*100*	*87*	*100*	*496*	*100*
Keine Angabe	1	–	4	–	3	–	7	–
**Gesamt**	**349**	**–**	**185**	**–**	**90**	**–**	**503**	**–**

Quelle: Lernen im Ausnahmezustand, Schüler_innenbefragung

## Footnotes

1 Der Datensatz ist über AUSSDA abrufbar. Dort findet sich auch eine genaue Beschreibung der Daten. Holtgrewe, Ursula; Vana, Irina; Lindorfer, Martina; Siller, Carmen. 2021. „Learning in a State of Emergency (SUF edition)“, 10.11587/PZHM0A, AUSSDA, V2, UNF:6:dK6NkTBBf50Gn3jOXPvx4A==[fileUNF].

2 Statistik Austria 2020b. Schülerstatistik nach Schultyp für Wien, Statcube, dl. 19.07.2022.

3 Berufe wurden mittels ISCO-Klassifikation kodiert, https://statistik.at/KDBWeb/kdb_VersionAuswahl.do.

4 Diese Variable wird in der PISA-Studie genutzt zur Annäherung an den Bildungsgrad der Familie (Mang et.al. 2021, S. 28). Fehlende Informationen zum Bildungsstatus der Eltern wurden mittels des durchschnittlichen Bücherbestands in Familien mit unterschiedlichen Bildungsabschlüssen geschätzt. Antworteten Eltern und Schüler_innen unterschiedlich, so wurden die Informationen der Eltern für die Klassifikation des Berufs herangezogen.

5 Der Abschluss der Eltern wurde nur im Elternfragebogen erhoben.

6 Kognitive Prozesse wurden nicht gemessen.

7 Diese werden bei AUSSDA als separates File zur Verfügung gestellt, 10.11587/PZHM0A.

8 Geprüft werden zumeist bivariate Verteilungen mittels X^2^. Für metrische Variablen mit zentralen Verteilungsmaßen (d. i. Indizes, die mittels Ordinaldaten mit einem Skalenniveau von mind. 5 berechnet wurden) werden Varianzanalysen (ANOVA) und Korrelationen berechnet.

9 Mit einem Wert von 0,21 von Cramers V zeigt sich bei Buben über 14 Jahren (*n* = 51) und Mädchen über 14 Jahren (*n* = 172) ein moderater, signifikant unterschiedener Zusammenhang (X^2^ = 10,0, df = 2, *p* = 0,00) hinsichtlich der Freude am Lernen im ersten Lockdown.

10 Insgesamt haben 231 Schüler_innen die Frage beantwortet. 35 davon stammten aus Familien mit niedrigem Bildungskapital. Im Verhältnis zu anderen Schüler_innen verdoppelt niedriges Bildungskapital die Chance, sich durch das Wegfallen des Klassenkontexts entlastet zu fühlen (X^2^ = 4,5, df = 1, *p* = 0,03).

11 Um zu erfassen, welche Faktoren Einfluss auf das Lernen zu Hause nehmen, wurde eine Korrelationsmatrix berechnet. Signifikante Zusammenhänge zeigten sich zwischen einem positiven Erleben des Lernens undder allgemeinen Zufriedenheit (r = 0,55, *p* = 0,00), „Ich komme gut mit den Aufgaben zurecht“ (r = 0,44, *p* = 0,00), „Ich kenne mich oft nicht aus“ (r = −0,41, *p* = 0,00) und „Ich habe oft keine Lust, die Aufgaben zu machen“ (r = 0,4, *p* = 0,00).

12 Gefragt wurde auf einer Skala von 1 (trifft sehr zu) bis 5 (trifft nicht zu): „Wie gut kommst du mit den Aufgaben zurecht?“. Über folgende Antworten wurde ein Mittelwertindex gebildet (positiv formulierte Fragen wurden umgepolt): „Ich kann die meisten Aufgaben gut erledigen“ (Ablehnung während des Lockdowns „sehr“ bzw. „eher“ 8 %); „Ich kenn mich oft nicht aus“ (Zustimmung „sehr“ bzw. „eher“ 18,2 %); „Mir fehlen oft Erklärungen“ (Zustimmung „sehr“ bzw. „eher“ 30,5 %). Cronbachs Alpha weist für die drei Variablen einen Wert von 0,816 und damit eine hohe interne Konsistenz auf. Abb. 2 zeigt den Anteil der Schüler_innen, die überwiegend ablehnten, dass sie mit den Aufgaben gut zurecht kamen (trifft nicht zu/trifft eher nicht zu); bzw. überwiegend zustimmten, dass sie Schwierigkeiten hatten (trifft sehr zu/trifft eher zu). D. i. Schüler_innen bei denen der Index einen Wert unter 3 aufwies.

13 Gefragt wurde auf einer Skala von 1 (nie) bis 5 (immer) wie häufig sich Schüler_innen im Lockdown überfordert fühlten. Ein signifikanter Unterschied besteht zwischen Schüler_innen aus einfach qualifizierten Familien und Schüler_innen aus akademischen Familien (X^2^ = 6,03, df = 2, *p* = 0,18).

14 Signifikante Unterschiede bestehen hinsichtlich der Frage „Ich versuche alleine die Lösung zu finden“ (Welch-Test F (3, 320) = 2,76, *p* < 0,04) mit folgenden Werten: einfach qualifiziert (X̅ = 3,94; SD = 1,03), mittel oder höher qualifiziert (X̅ = 3,61; SD = 1,08), hoch qualifiziert (X̅ = 3,47; SD = 1,09); und bei der Frage „Ich frage meine Mutter oder meinen Vater, ob sie mir helfen können“ (Welch-Test F (2, 112,4) = 12,5, *p* < 0,000) mit folgenden Werten: einfach qualifiziert (X̅ = 1,89; SD = 1,20), mittel oder höher qualifiziert (X̅ = 2,46; SD = 1,38); hoch qualifiziert (X̅ = 3,13; SD = 1,43). Die Unterschiede zwischen den Mittelwerten der Gruppen bei der Frage, ob Lehrkräfte und/oder Mitschüler_innen helfen, sind nicht signifikant und werden daher hier nicht angeführt.

15 212 Schüler_innen antworteten auf die Frage.

16 Die Fallzahlen nach Bildungskapital sind gering, weshalb Unterschiede nur als Trend zu werten sind. Ein signifikanter Unterschied besteht zwischen Schüler_innen einfach und mittel bzw. höher qualifizierten Familien und Schüler_innen aus akademischen Familien beim Wunsch nach technischer Ausstattung zuhause (X^2^ = 7,13, df = 2, *p* = 0,23.).

17 Anteil der Schüler_innen, die eine Veränderung als sehr bzw. eher wünschenswert bezeichneten.
